# Comment on *"*IGF2BP3 promotes the proliferation and cisplatin resistance of bladder cancer by enhancing the mRNA stability of CDK6 in an m^6^A dependent manner*"*

**DOI:** 10.7150/ijbs.120024

**Published:** 2025-08-16

**Authors:** Yongzheng Han, Chengming Li, Guangzhen Wu

**Affiliations:** Department of Urology, the First Affiliated Hospital of Dalian Medical University, Dalian, 116011, China.

We are writing to commend the recent article by Song *et al.*, titled "***IGF2BP3 promotes the proliferation and cisplatin resistance of bladder cancer by enhancing the mRNA stability of CDK6 in an m^6^A dependent manner*** 1," published in the ***International Journal of Biological Sciences***. This study demonstrates that Insulin-like growth factor 2 mRNA binding protein 3 (IGF2BP3) promotes bladder cancer (BCa) proliferation and chemoresistance in an m^6^A-dependent manner by directly stabilizing Cyclin-dependent Kinase 6 (CDK6) mRNA. Palbociclib effectively reverses this phenotype. By comparing the mechanisms of IGF2BP3 in other tumors, such as IGF2BP3 targeting PD-L1 in breast cancer 2, our commentary highlights the unique role of the IGF2BP3/m^6^A/CDK6 axis in BCa. We also provide a broader context for its oncogenic functions. In addition to CDK6, m^6^A modification plays a key role for various major members of the CDK family. IGF2BP3 increases the stability of CDK2 in an m^6^A-dependent manner and affects the proliferation of BCa cells 3, while FTO-mediated m^6^A demethylation stabilizes CDK2 mRNA 4. These findings suggest a broader regulatory role for m^6^A in CDK members.

FTO, functioning as an m^6^A demethylase, drives BCa cell proliferation through the FTO/miR-576/CDK6 pathway 5. Song *et al.* highlight IGF2BP3's role in stabilizing CDK6 mRNA, suggesting its potential involvement in m^6^A-related pathways. FTO modulates several signaling axis implicated in BCa progression, including the FTO/MALAT axis 6 and FTO/STAT3 axis 7. Furthermore, IGF2BP3 in glioma promotes FTO degradation through the ubiquitin-proteasome pathway , contributing to therapy resistance 8. Future research should explore the role of IGF2BP3 in other m^6^A-related signaling pathways. For example, the FTO/m^6^A/MYC axis, which is implicated in diverse tumor types. Elucidating these interactions could advance oncology therapeutics.

IGF2BP3 expression correlates positively with inflammation and immune infiltration in BCa. Mechanistically, IGF2BP3 stabilizes HMGB1 mRNA, thereby upregulating its expression 9. This finding indicates that IGF2BP3 may promote BCa progression and immune infiltration through multiple pathways. Future studies could explore the potential crosstalk among IGF2BP3-related signaling pathways. Additionally, it is valuable to investigate whether the IGF2BP3/m^6^A/CDK6 axis influences the tumor microenvironment. Elucidating these mechanisms may identify novel treatment strategies for BCa patients.

Palbociclib demonstrates therapeutic potential to overcome cisplatin resistance in BCa. However, significant hematologic toxicity remains a consistent clinical concern 10. While this study highlights the efficacy of palbociclib, the organ-specific toxicity of cisplatin-palbociclib combination therapy requires further investigation. Its application in the clinic requires careful evaluation of long-term safety and systemic effects (Figure 1).

Song *et al.* establish the IGF2BP3/m^6^A/CDK6 axis as a treatment target in BCa. Further studies may elucidate the role of RNA modifications in chemotherapy resistance and drive clinical translation.

Sincerely.

## Figures and Tables

**Figure 1:**
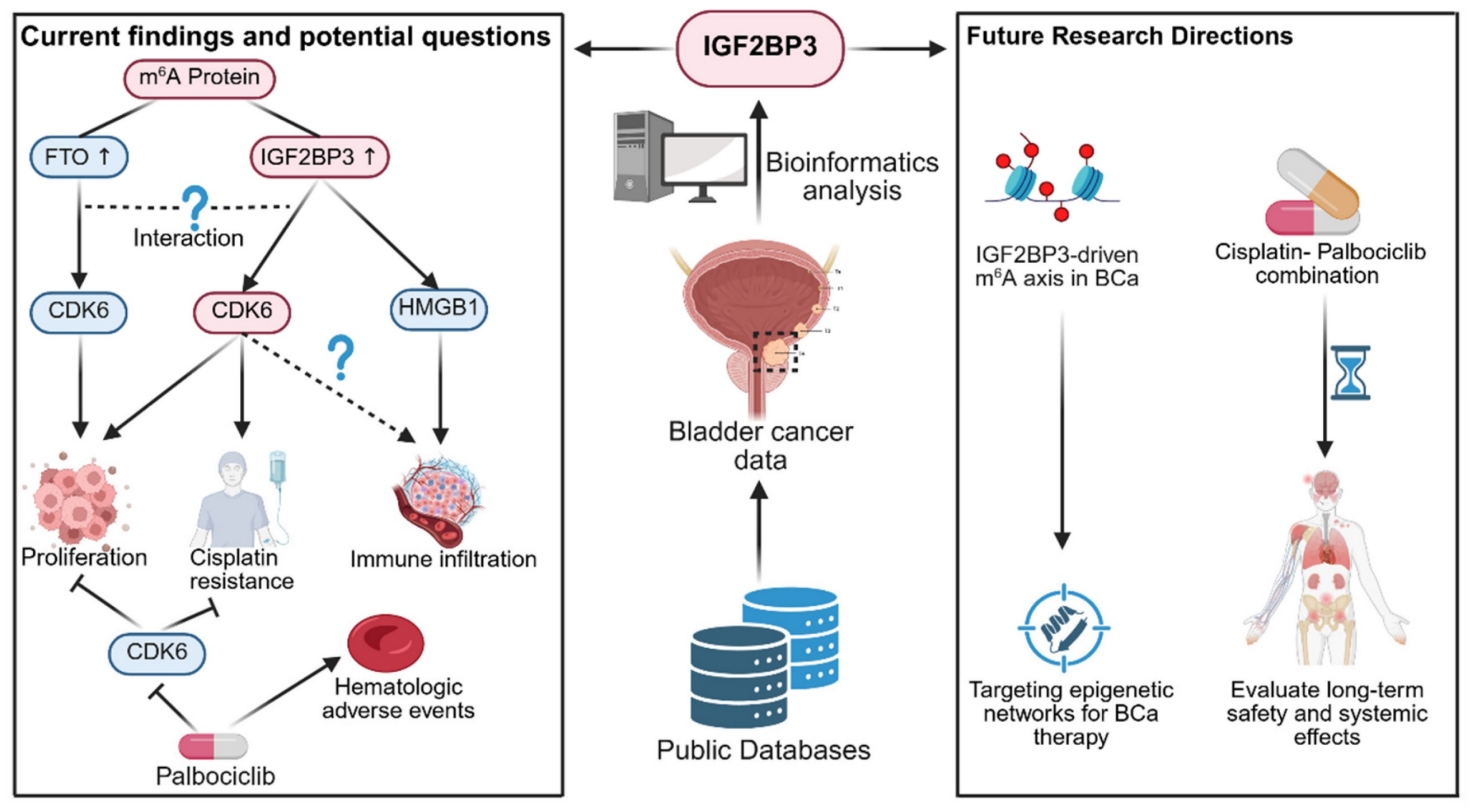
** An overview of current findings and potential questions in BCa.** This figure highlights how IGF2BP3 interacts with RNA modification proteins to influence proliferation, cisplatin resistance, and immune infiltration. On the right, directions for future research are proposed.

## Abbreviations

BCa: Bladder Cancer
CDK2: Cyclin-dependent Kinase 2
CDK6: Cyclin-dependent Kinase 6
FTO: Fat mass and obesity associated protein
HMGB1: High mobility group box 1
IGF2BP3: Insulin-like growth factor 2 mRNA binding protein 3
MALAT: Metastasis-associated lung adenocarcinoma transcript 1
m^6^A: N6-methyladenosine
miR-576: MicroRNA-576
MYC: MYC proto-oncogene
CDK6: Cyclin-dependent Kinase 6
PD-L1: Programmed death-ligand 1
STAT3: Signal transducer and activator of transcription 3
